# Metabolic Fingerprinting Uncovers the Distinction Between the Phenotypes of Tuberculosis Associated COPD and Smoking-Induced COPD

**DOI:** 10.3389/fmed.2021.619077

**Published:** 2021-05-14

**Authors:** Da Jung Kim, Jee Youn Oh, Chin Kook Rhee, Seoung Ju Park, Jae Jeong Shim, Joo-Youn Cho

**Affiliations:** ^1^Department of Clinical Pharmacology and Therapeutics, Seoul National University College of Medicine and Hospital, Seoul, South Korea; ^2^Seoul National University Medical Research Center, Seoul National University College of Medicine and Hospital, Seoul, South Korea; ^3^Division of Pulmonary, Allergy and Critical Care Medicine, Department of Internal Medicine, Korea University Guro Hospital, Seoul, South Korea; ^4^Division of Pulmonary Medicine, Department of Internal Medicine, Catholic University Seoul Hospital, Seoul, South Korea; ^5^Division of Pulmonary Medicine, Department of Internal Medicine, Chonbuk National University Hospital, Jeonju, South Korea; ^6^Department of Biomedical Sciences, Seoul National University College of Medicine, Seoul, South Korea

**Keywords:** chronic obstructive pulmonary disease, tuberculosis, metabolomics, biomarker, smoking

## Abstract

**Background:** Although smoking is considered the main cause of chronic obstructive pulmonary disease (COPD), several other risk factors, including pulmonary tuberculosis (TB), contribute significantly to disease causation, particularly in developing countries. However, the underlying pathogenesis of TB-associated COPD (T-COPD) is unclear. Moreover, the need for prompt diagnosis and treatment of T-COPD to decrease the future burden of inflammation is underestimated. This study aimed to identify distinctive endogenous metabotypes of T-COPD, compared to smoking-associated COPD (S-COPD).

**Methods:** Cross-sectional metabolomic analyses and clinical examinations of serum samples were performed for three groups of 168 male subjects: T-COPD (*n* = 59), S-COPD (*n* = 70), and healthy normal controls (*n* = 39). To retain a broad spectrum of metabolites, we performed technically distinct analyses (global metabolomic profiling using LC-QTOFMS and targeted analyses using LC-MS/MS).

**Results:** Higher levels of IL-6 and C-reactive protein and St. George Respiratory Questionnaire scores were seen in the T-COPD group, compared to those in the S-COPD group. Global metabolomic profiling showed elevated metabolites, including arachidonic and eicosanoic acids, in the T-COPD group. Typical changes in tryptophan catabolism were observed through targeted profiling. Additionally, in the T-COPD group, kynurenine was elevated, and serotonin levels were reduced; therefore, indoleamine dioxygenase (IDO)/tryptophan hydroxylase (TPH) activities were dysregulated. Correlation analyses showed that changes in oxylipins were positively correlated with serum levels of IL-6 and C-reactive protein.

**Conclusion:** Patients with TB-related COPD have enhanced inflammatory responses that may be linked to fatty acid pathways and tryptophan catabolism, which could be novel therapeutic targets for T-COPD.

## Introduction

Chronic obstructive pulmonary disease (COPD) is a major global health problem that causes airflow limitation and increases patient vulnerability to exacerbations and serious inflammatory responses (1). Apart from smoking, several other risk factors, including biomass fuel smoke exposure, childhood lower respiratory tract infections, outdoor air pollution, and treated cases of pulmonary tuberculosis (TB) play a significant role in disease etiology, particularly in developing countries (2). Approximately 10 million people are infected with TB annually, and pulmonary system injury is the major effect of TB (3). Appropriate treatment can effectively cure TB. However, permanent COPD occurring after eradication of the organism is seen in more than 50% of the infected patients (3, 4). TB-associated COPD (T-COPD) develops at a younger age compared with smoking-associated COPD (S-COPD) (5–7). However, prompt diagnosis and treatment of T-COPD based on an understanding of the pathogenesis to decrease the future burden of chronic airflow obstruction and inflammation are seldom achieved (8).

T-COPD was previously considered a static disease causing only parenchymal destruction, and extensive research on the treatment and control of morbidities in these patients have not been conducted to the same extent as with S-COPD. However, lung remodeling and chronic inflammatory responses have recently been shown to persist in these patients after treatment completion (9, 10). The pathogenesis and inflammatory properties of T-COPD need to be investigated and compared with those of S-COPD, to evaluate the need for the treatment and management of T-COPD.

Global profiling of metabolites in biofluids and tissues has been popularized for identifying the underlying mechanisms, developing disease-specific or grade-specific biomarkers, and assessing therapeutic effects (11–13). The identification of numerous endogenous metabolites has been used to characterize patients with pulmonary diseases, and endogenous metabotyping is a useful technique to differentiate the characteristic features of diseases with very similar phenotypes, such as asthma and chronic airway obstructive disease (11, 14). In this study, we used metabolomics to characterize patients with S-COPD and T-COPD, and we investigated the associations of various metabolites with these disease groups.

## Methods

### Study Population

This multi-center cohort study was performed to identify clinically useful biological markers in chronic airway diseases for the diagnosis and assessment of treatment response, conducted at the Korea University Guro Hospital, Seoul St. Mary's Hospital, and Chonbuk National University Hospital. The study protocol was approved by the institutional review boards (Korea University Guro Hospital: KUGH 13246; Seoul St. Mary's Hospital: KC15OIMI0553; and Chonbuk National University Hospital: 2015-01-018-005), and it fulfilled the tenets of the Declaration of Helsinki. The patients provided written informed consent.

The inclusion criteria for patients with T-COPD were: (1) history of TB with no change in the chest images over the past year; (2) at least one finding of destroyed pulmonary parenchyma on chest images (lung volume loss, bronchovascular distortion, fibrosis, or secondary bronciectasis), with the total volume of all lesions being greater than one-third of one lung, confirmed by a radiologist or pulmonologist; (3) airflow limitation (post-bronchodilator spirometry with forced expiratory volume in 1 second [FEV_1_]/forced vital capacity [FVC] < 70%) and no history of asthma or COPD before the diagnosis of TB; and (4) no history of respiratory infections within the previous 6 weeks.

S-COPD was diagnosed according to the American Thoracic Society and Global Initiative for Chronic Obstructive Lung Disease guidelines (post-bronchodilator spirometry with FEV_1_/FVC < 70%). Patients with a smoking history of > 10 packs per year were enrolled, and patients with bronchiectasis, sequelae from a prior TB infection, interstitial lung disease, or other diseases causing airflow obstruction, were excluded.

Blood samples and data from healthy normal controls (NC) with no history of COPD or respiratory symptoms, who had visited for general health assessments, were provided by the Biobank of Korea University Guro Hospital (KU Guro Gene Bank 2018-026). Data on age, sex, body mass index (BMI), and smoking status were collected. For patients with S-COPD and T-COPD, the variables collected at baseline were smoking amount, BMI, symptom scores, results of pulmonary function tests, rate of decline in FEV_1_, acute exacerbation history, and chest imaging data. Detailed protocol of measurements of clinical parameters is available in the Supplementary Material.

### Global Metabolomics Profiling Using LC-QTOF-MS

The frozen samples of plasma and urine were briefly diluted with an acetonitrile:methanol:water (3:3:4) mixture (HPLC-grade, Millipore, Bedford, MA, USA) and 10% methanol, respectively. Each sample (5 μL) was loaded onto an Acquity UPLC BEH C18 (1.7 μm, 2.1 mm × 100 mm; Waters Corp., Milford, MA, USA) column and analyzed using an Agilent 6530 QTOF mass spectrometer (Agilent Technologies). The experimental procedure is described in detail in our previous studies (12, 15). All samples were examined simultaneously in a single run to avoid the batch effect and the overall quality of the analysis procedure was monitored using repeat extracts of a pooled plasma sample.

### Targeted Metabolomics Using LC-MS/MS

The AbsoluteIDQ^®^ p180 kit with an LC-MS/MS system was used to quantify 188 metabolites, allowing the concurrent high-throughput detection and quantification of metabolites in serum samples. The sample preparation and LC-MS/MS analytical procedure are described in detail in our previous reports (13). The average % coefficient of variance of analytes ranged between 15–30 % CV range and the concentrations of limit of detection are listed in Supplementary Table 1.

### Statistical Analysis

Clinical data were presented as the median and interquartile range for continuous variables and as a percentage for categorical variables. Data were compared using the Mann–Whitney *U*-test or Kruskal–Wallis test for continuous variables, and Pearson's χ^2^ test or Fisher's exact test for categorical variables. ANCOVA was used to compare age, BMI, smoking and underlying diseases for IL-6 and CRP. Statistical significance was defined as *P* < 0.05. All statistics were analyzed using SPSS 20.0 software (IBM, Chicago, Illinois, USA). We performed logistic regression of discrimination of T-COPD and S-COPD for targeted metabolites. Variables with a *P* < 0.05 on univariate analysis and demographic factors including age, BMI, underlying diseases and smoking history were analyzed using multivariate logistic regression analysis. ROC analysis was done for significant metabolites by multivariate logistic regression.

Statistical analyses for the untargeted and targeted metabolic data and network analysis for visualization are available in the Supplementary Material.

## Results

### Patient Demographics

The study included 168 patients (T-COPD, *n* = 59; S-COPD, *n* = 70; NC, *n* = 39). The median age of NCs was 55.0 years (50.0–65.0 years), and the median BMI, 23.7 (22.3–26.1) (Supplementary Table 2). Table 1 shows the baseline characteristics of patients with T-COPD and S-COPD. The patient-reported scores for symptoms and quality of life were similar in both groups, except for the St. George Respiratory Questionnaire (SGRQ) score, which was higher in the T-COPD group than in the S-COPD group. The rate of FEV_1_ decline and the percentage of acute exacerbations did not show significant differences among the groups. C-reactive protein (CRP) levels, erythrocyte sedimentation rate (ESR), and IL-6 levels were significantly higher in patients with T-COPD than in those with S-COPD (Table 1).

**Table 1:** Demographics of patients with S-COPD and T-COPD.

	**S-COPD (*N* = 70)**	**T-COPD (*N* = 59)**	***P*-value**
Age, years	66.0 (59.0–72.5)	68.0 (64.0–73.0)	0.165
Sex, male	70 (100)	59 (100)	–
BMI, kg/m^2^	23.1 (21.1–25.9)	22.9 (21.1–25.5)	0.519
Smoking amount (pack-year)	40.0 (30.0–50.0)	0.0 (0.0–30.0)	<0.001
**Comorbidities**
Heart disease	27 (38.6)	25 (42.4)	0.661
Cerebrovascular disease	9 (12.9)	5 (8.5)	0.425
Depression	2 (2.9)	1 (1.7)	0.663
**Pulmonary function tests**			
FEV_1_ % predicted	62.5 (45.8–71.0)	54.0 (45.0–73.0)	0.496
FVC % predicted	84.0 (74.0–93.3)	78.0 (66.0–89.0)	0.089
FEV_1_/FVC % predicted	54.5 (41.0–62.0)	53.0 (42.0–65.0)	0.465
DL_CO_ %	80.5 (64.8–97.0)	75.0 (62.0–87.0)	0.180
**Symptom scores**			
mMRC	1.0 (0.0–1.0)	1.0 (0.0–1.0)	0.654
CAT	14.0 (10.0–19.0)	17.0 (11.0–21.0)	0.096
SGRQ	16.9 (9.0–29.2)	22.1 (13.8–34.5)	0.048
FEV_1_ decline ml/yr	0.5 (-50.0–170.0)	60.0 (-30.0–170.0)	0.335
Frequent AE *n*, (%)	7 (10.0%)	12 (20.3%)	0.099
**Inflammatory markers**			
IL-6 (pg/dl)	1.46 (0.43–2.46)	2.67 (1.52–6.80)	<0.001^*^
ESR (mm/h)	10.00 (6.00–16.25)	15.00 (7.00–21.00)	0.011^*^
CRP (mg/dl)	1.25 (0.63–2.30)	3.39 (1.49–6.74)	0.044^*^

*Data are presented as median (interquartile range) for continuous variables and number (percentage) for categorical variables*.

* *This P-value was analyzed by ANCOVA (after adjusting age, BMI, smoking, and underlying diseases)*.

*AE, acute exacerbation; BMI, body mass index; CAT, chronic obstructive pulmonary disease assessment test; CRP, C-reactive protein; ESR, erythrocyte sedimentation rate; DL_CO_, diffusing capacity of the lung for carbon monoxide; FEV_1_, forced expiratory volume in 1 s; FVC, forced vital capacity; IL-6, interleukin-6; mMRC, modified Medical Research Council; S-COPD, smoking-induced chronic obstructive pulmonary disease; SGRQ, St. George Respiratory Questionnaire; T-COPD, tuberculosis-associated COPD*.

### Metabotyping of Patients: Global and Targeted Metabolic Profiling

**Figure 1:**
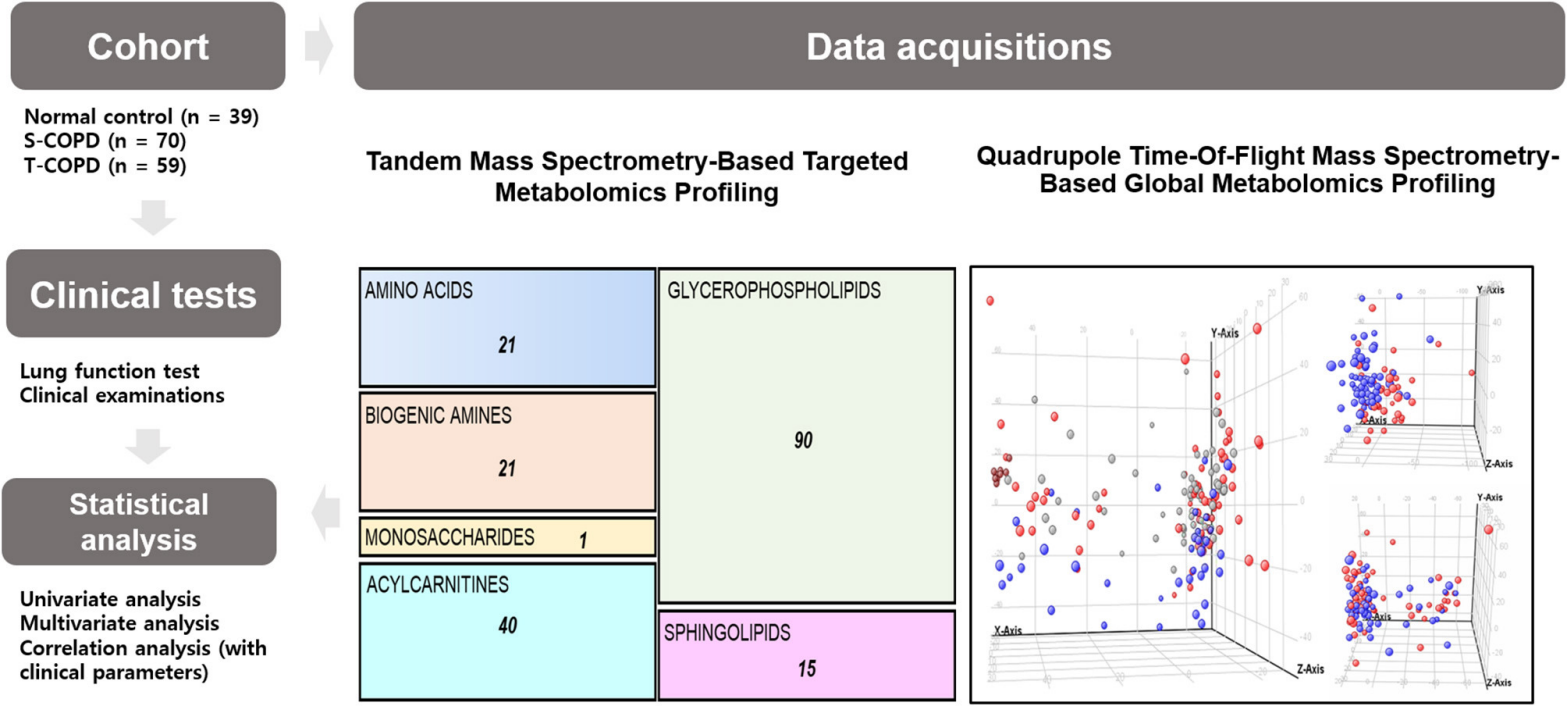
Schematic of the study. This study included 39 healthy subjects, 70 patients with S-COPD, and 59 patients with T-COPD. All patients underwent clinical examinations, including lung function tests and metabolic profiling. For the targeted analysis, a tandem mass spectrometry-based technique was used. The absolute quantification of 188 metabolites, including 21 amino acids, 21 biogenic amines, one monosaccharide, 40 acylcarnitines, 90 glycerophospholipids, and 15 sphingolipids, was performed. For global metabolomic profiling, a QTOF-MS-based technique was chosen. Metabolic biomarkers were selected by performing multivariate and univariate analyses. The correlations of the metabolic biomarkers with clinical parameters were then calculated. NC, normal control; T-COPD, TB-associated COPD; S-COPD, smoking-associated COPD.

**Figure 2:**
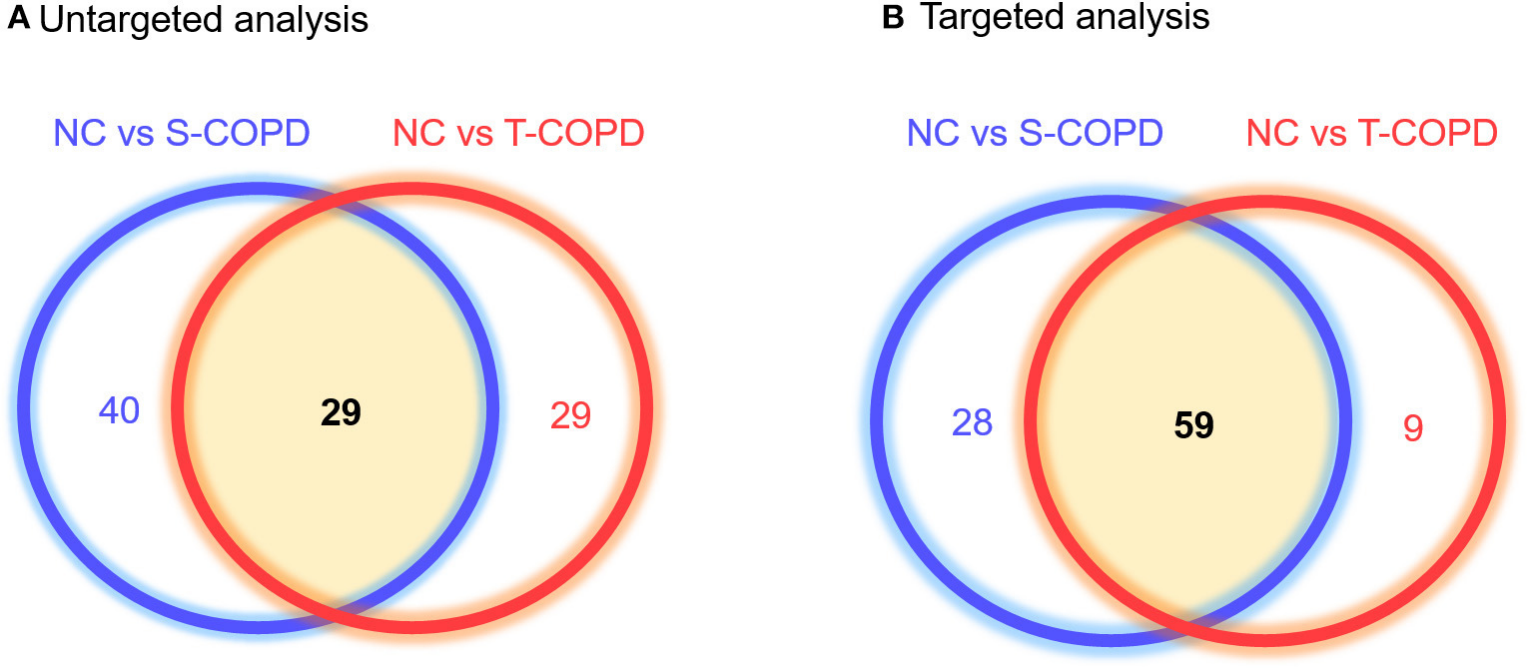
Venn diagram of metabolites identified in patients with T-COPD and S-COPD. **(A)** Comparison of the NC and S-COPD groups revealed that 8,183 ions and 69 metabolites were significantly altered. In the comparison of the NC and T-COPD groups, 5,193 ions were detected, and 58 metabolites were significantly different. Twenty-nine metabolites were shared by the two COPD groups. **(B)** Using the targeted analysis, 188 metabolites were quantified, 87 metabolites were significantly altered between the NC and S-COPD groups, and 68 metabolites were significantly altered between the NC and T-COPD groups. Fifty-nine metabolites overlapped in these two comparisons and their direction of regulation was also similar in the S-COPD and T-COPD groups, compared to the NC group. The blue circle indicates metabolic biomarkers obtained from the comparison of the NC and S-COPD groups, and the red circle represents metabolic biomarkers identified in the comparison of the NC and T-COPD groups. NC, normal control; T-COPD, TB-associated COPD; S-COPD, smoking-associated COPD.

The metabolic profiling workflow is shown in Figure 1. We used two different techniques to obtain a broad range of candidate metabolites. We performed global metabolomics profiling, which can screen thousands of unknown features in two conditions. The detected metabolic features were then subjected to statistical analyses. Supplementary Figure 1 shows the results of the unsupervised pattern recognition analysis. Supplementary Figures 1A–C shows the score plots describing variation and account for the varied influences of the NC and S-COPD groups, the NC and T-COPD groups, and the T-COPD and S-COPD groups. In the univariate analysis, FDR-adjusted values <0.05 with a 2.0-fold change were applied for marker selection. Sixty-nine metabolites remained after the univariate analysis compared the NC and S-COPD groups, and 58 metabolites remained after the comparison for the NC and T-COPD groups. Among these, 29 metabolites overlapped and their changes (increase or decrease) showed similar trends (Figure 2A). Volcano plots of each analysis are available in Supplementary Figure 2.

For further analysis, targeted metabolic profiling was performed, which measures the absolute concentrations of molecules and the conversion rates from one molecule to another. For the targeted analysis, 188 metabolites were quantified based on the LC-MS analyses. This technique provided quantitative data for 40 acylcarnitines, 42 amino acids, biogenic amines, monosaccharides, 15 sphingolipids, and 90 glycerophospholipids in each sample. The absolute concentration in each sample was then adjusted for statistical analysis, with a *P*-value <0.05, indicating significance. We obtained 87 distinct metabolites that differentiated the NC and S-COPD groups, and 68 metabolites that differentiated the NC and T-COPD groups (Figure 2B).

**Figure 3:**
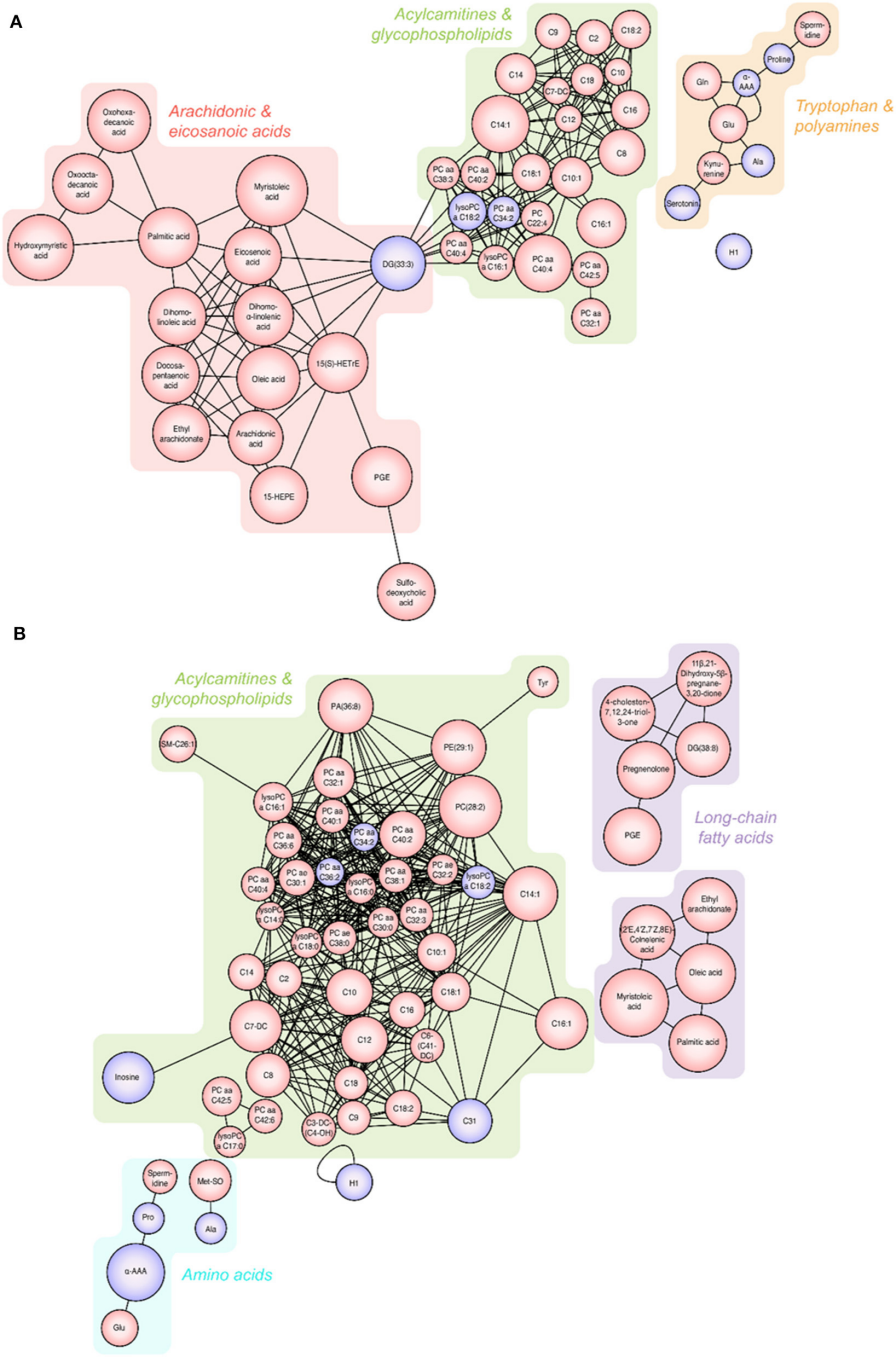
Changes in metabolites seen in patients with T-COPD and S-COPD. **(A)** Significantly altered metabolites were observed between the NC and T-COPD groups. **(B)** Significantly altered metabolites were observed between the NC and S-COPD groups. The size of the circle indicates the relative fold change compared to the NC group. The red circle represents increased levels and the purple circle represents decreased levels of metabolites in patients, compared to healthy controls. NC, normal control; T-COPD, TB-associated COPD; S-COPD, smoking-associated COPD.

Significantly altered metabolites are displayed in Figure 3. All the identified metabolites were mapped and linked to each other based on their biological interactions. In patients with S-COPD, we observed increased levels of long-chain fatty acids (specifically myristoleic acid, palmitic acid, and oleic acid), bile acid-related metabolites, and multiple glycerophospholipids and acylcarnitines (Figure 3A and Supplementary Table 3). All metabolites identified using global metabolomics are listed in Supplementary Table 4. In the T-COPD group, we observed specific changes in the arachidonic and eicosanoic acid metabolic pathways (Figure 3A and Supplementary Table 3). Similar changes in the levels of myristoleic acid, palmitic acid, oleic acid, ethyl arachidonate, and prostaglandin E, were observed in all the groups. However, there was more than a two-fold increase in the levels of arachidonic acid, eicosanoic acid, docosapentaenoic acid, dihomo-alpha-linolenic acid, dihomo-linoleic acid, hydroxyeicosatrienoic acid (HETrE), and hydroxyeicosapentaenoic acid (HEPE) in the T-COPD patients, compared to the healthy controls, whereas no gradual changes were observed in the S-COPD group.

### Changes in Tryptophan Catabolism

**Figure 4:**
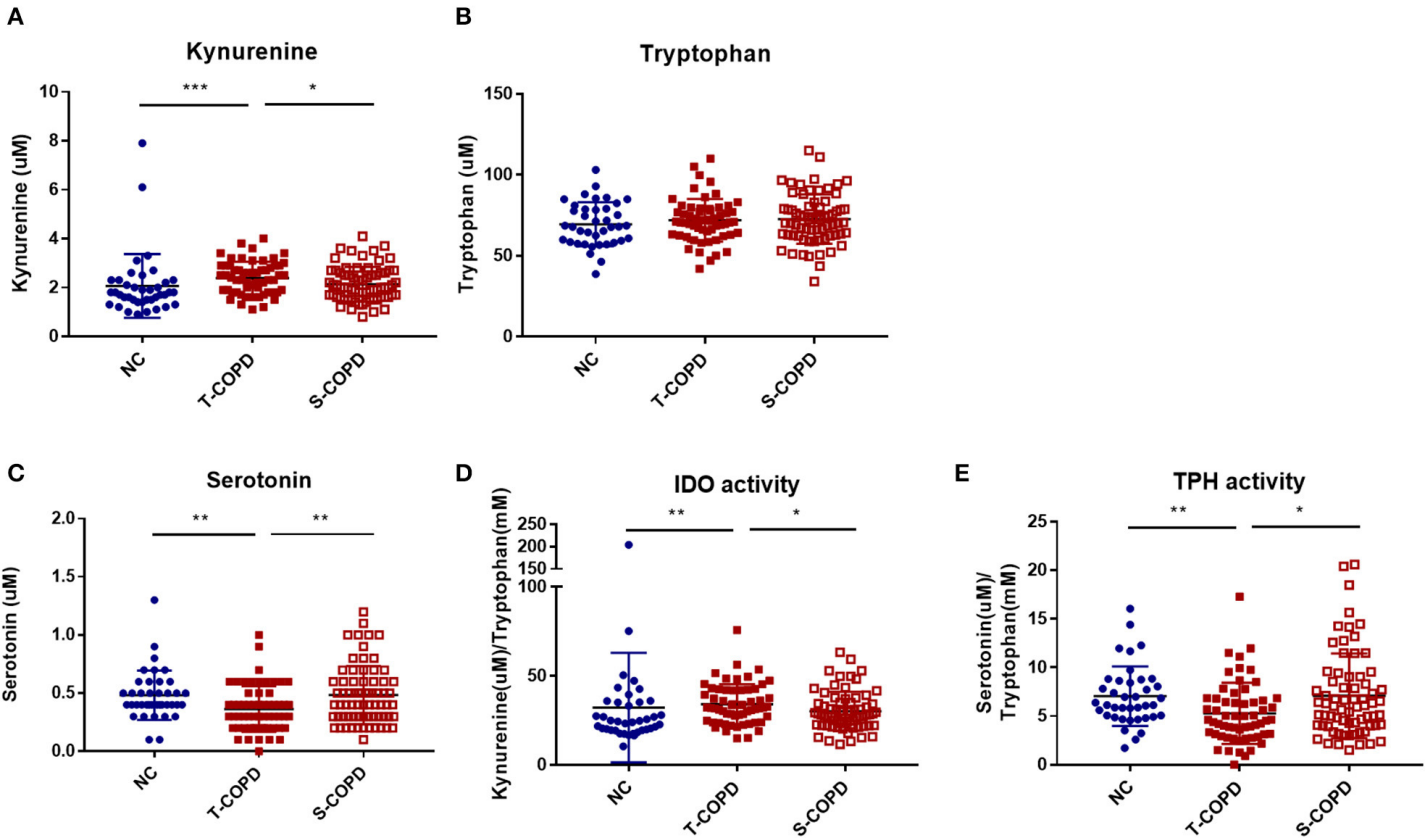
Alternation of tryptophan metabolism in patients with T-COPD. Serum concentrations of **(A)** kynurenine, **(B)** tryptophan, and **(C)** serotonin are shown in scatter plots. **(D)** and **(E)** indicate IDO and TPH activities, respectively. Data are presented as the mean ± standard error of the mean. ^*^P-value <0.05, ^**^P-value < 0.01, and ^***^P-value < 0.001. NC, normal control; T-COPD, TB-associated COPD; S-COPD, smoking-associated COPD; IDO, indoleamine dioxygenase; TPH, tryptophan hydroxylase.

**Figure 5:**
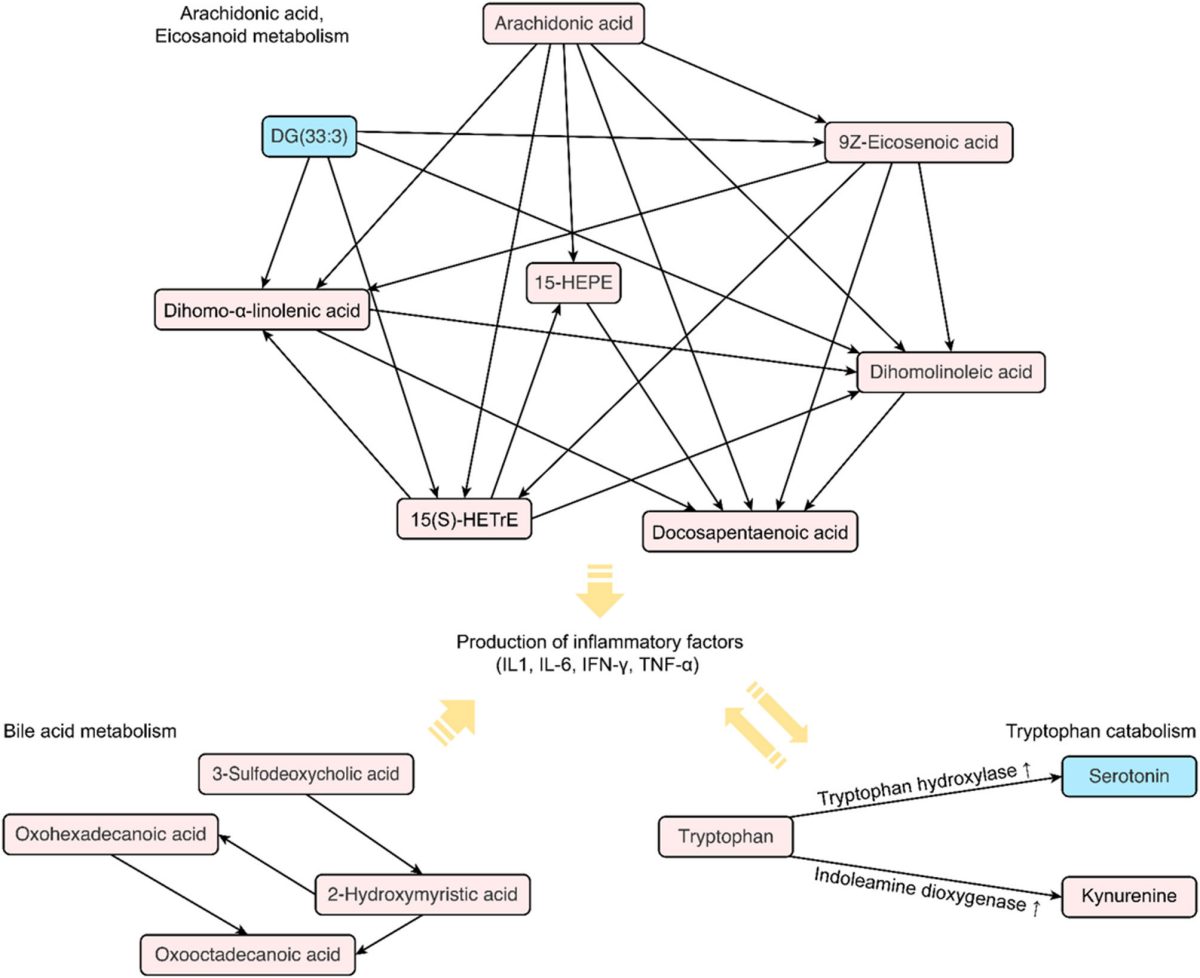
Distinct metabolic pathways identified in patients with COPD following a TB infection. The diagram shows a map of metabolic biomarkers exclusively selected in patients with TB-induced COPD. The red square indicates increased levels of metabolites in the serum of patients with COPD, whereas blue indicates decreased levels. The arrow indicates the possible direction of the metabolic pathway based on the KEGG database.

Among the 188 targeted metabolites subjected to absolute quantification, 59 were shared among the COPD groups and predominantly comprised acylcarnitines or glycerophospholipids. Notably, biogenic amines (kynurenine and serotonin) and amino acids (aspartate and threonine) were detected in the T-COPD group. Furthermore, the association between tryptophan catabolism and disease status was investigated (Figure 4). Although the total serum concentration of tryptophan did not change among the groups, we observed significant alterations in the kynurenine and serotonin levels in T-COPD patients (Figures 4A,C). Along with the changes in metabolite levels, the enzymatic activities of indoleamine dioxygenase (IDO) and tryptophan hydroxylase (TPH) were altered in the T-COPD group, which showed increased IDO activity compared with the control group (Figure 4D), and decreased TPH activity (Figure 4E). These changes were reversed in the S-COPD group. Although the total tryptophan level did not change, tryptophan catabolizing enzyme activities differed according to disease status, particularly in the T-COPD group. A categorical analysis was subsequently performed to determine whether the levels of these metabolites changed with disease severity (Supplementary Figure 3). Patients in each group were divided based on a FEV_1_ cut-off value of 50%. None of these markers, including tryptophan, kynurenine, serotonin, and IDO/TPH activity, were related to lung function. Thus, these metabolic biomarkers of T-COPD are unique phenotypes in the COPD population; however, they still have a limited ability to identify disease severity. Figure 5 reveals the distinct metabolic pathways that were specifically altered in patients with T-COPD. After excluding the metabolites that overlapped with those identified in patients with S-COPD, metabolic pathways related to arachidonic and eicosanoic acid, bile acid metabolism, and tryptophan catabolism, were identified in this study.

### Discrimination of T-COPD and S-COPD by Targeted Metabolites

We further performed logistic regression of discrimination of T-COPD and S-COPD for targeted metabolites. Aspartate was the important metabolites associated with T-COPD diagnosis and serotonin and asparagine were the important metabolites associated with S-COPD (Supplementary Table 5). When we perform ROC analysis with aspartate, asparagine and serotonin, the AUC of T-COPD diagnosis was 0.780 (Supplementary Figure 4).

## Discussion

This is the first study to comprehensively profile serum samples from patients with COPD caused by smoking or a previous TB infection. The levels of IL-6 and CRP, and the SGRQ scores, were found to be higher in patients with T-COPD than in those with S-COPD. The multi-platform approach further discovered endogenous metabolites that distinguished these two disease groups. The metabolism of fatty acids, especially arachidonic acid and eicosanoic acid, and tryptophan catabolism were correlated, particularly in the T-COPD group. These results are consistent with the findings of our previous study, demonstrating an association between systemic inflammation and the COPD sub-phenotype in this cohort (16).

Overall, the metabolites identified in patients with T-COPD and S-COPD shared some common features, as shown in Figure 2. The presence of similar physiological symptoms such as dyspnea, sputum production, and acute exacerbation might result in similar metabolic changes, even though the etiology of S-COPD and T-COPD are different. We observed major changes in the levels of acylcarnitines and phospholipids in both COPD groups, with the levels being higher than that in healthy subjects. The close relationship between acylcarnitines and physiological activities is well-known as the evidence of energy metabolism. Circulating fatty acids are generally conjugated with carnitines to be utilized as an energy source in mitochondria, and their use is decreased in damaged lungs (17). Thus, increased serum acylcarnitine levels can be expected in COPD, and altered phospholipid levels could be another metabolic hallmark of COPD. The imbalance between phospholipids and lysophospholipids in COPD is controversial. However, multiple studies have reported a close association between increased levels of circulating phospholipids and the production of proinflammatory mediators during COPD exacerbations (18).

Changes in fatty acid metabolites indicate notable distinction between T-COPD and S-COPD. Here, the T-COPD group showed higher levels of some lipid metabolites, such as arachidonic acid or eicosanoic acids, than the S-COPD group did. Arachidonic acid, a polyunsaturated fatty acid (PUFA), is a member of the ω-6 fatty acid family and the primary target of cyclooxygenases (COX) and lipoxygenase (LOX). By-products such as prostaglandins and eicosanoids, well-known proinflammatory mediators, are generated following oxidization (17). Lipid metabolism in the lungs is associated with chronic respiratory failure, airway obstruction, bronchoconstriction, IL-6 generation, vascular permeability, airway remodeling, mucus secretion, and inflammation in COPD (19). Interestingly, excess levels of arachidonic acid and eicosanoids were observed in the T-COPD group. Additionally, oxylipins were positively correlated with IL-6 and CRP levels, and docosapentanoic acid, 15-HEPE, and long-chain fatty acids were associated with worsening lung function and acute exacerbation. As lipid metabolite levels were increased in both disease groups compared to the controls, being significantly higher in the T-COPD group than in the S-COPD group, T-COPD can be considered to have similar pathogenesis as S-COPD, but causes more severe local and systemic inflammation.

Moreover, tryptophan catabolism showed greater activation in the T-COPD group than in the S-COPD group. Tryptophan catabolism through the IDO pathway exerts antimicrobial and anti-inflammatory effects in certain infectious or inflammatory conditions (20, 21). Increased levels of inflammatory cytokines such as IL-6 induce IDO expression, and IDO induces regulatory T cells to regulate inflammatory responses (22). The effect of tryptophan catabolism on inflammation is known; several studies have already analyzed IDO and TPH activities in COPD patients. Although a study reported increased IDO activity during acute exacerbations of COPD (21), no apparent alterations in this biological process have been reported to date (23, 24). Similar to the results from previous studies, the levels of the associated metabolites were altered in the T-COPD group, but not in the S-COPD group. Furthermore, higher systemic levels of inflammatory mediators, such as IL-6 and CRP, were observed in the T-COPD group than in the S-COPD group. Based on these findings, coexisting bronchiectasis, destroyed lung parenchyma, and bacterial colonization may cause repeated infections and inflammation (25, 26). Activation of immunological mechanisms may also result in sustained systemic inflammation after TB, and elevated IL-6 levels may subsequently induce activation of the IDO pathway.

The contribution of the gut microbiota cannot be excluded. Tryptophan is a microbiome-derived amino acid catalyzed by three pathways: (1) direct conversion into ligands of the aryl hydrocarbon receptor via the gut microbiota; (2) entry into the IDO-mediated kynurenine pathway in immune and epithelial cells; and (3) serotonin production by tryptophan hydroxylase in enterochromaffin cells (27, 28). Although the role of functional microbiota in the pathophysiology of COPD is being investigated, alterations in the gut microbiome have been observed; its diversity decreases with increasing disease severity, compared to healthy subjects (29, 30). Nevertheless, the relationship between tryptophan catabolism and COPD is controversial, although the role of TB infection in tryptophan catabolism is well-known. According to previous *in vivo* and *in vitro studies*, TB induces potent activation of IDO, thereby inducing tryptophan catabolism for kynurenine production, which predominantly occurs in lung and immune cells (29, 31). IDO activity suppression has been shown to improve clinical symptoms of lung destruction and immune response in experimental studies (29). Although T-COPD patients were completely cured of TB, there could have been some irreversible continuous pathophysiological changes. Therefore, these patients might experience persistent effects of the resolved TB infection throughout their lifetime.

Smoking increases systemic neutrophil infiltration and sequesters leukocytes in lung capillaries by decreasing their deformability (32). Factors directly modulating the host immune system increase the production of inflammatory mediators such as interleukins, tumor necrosis factor-alpha (TNF-α), and CRP, resulting in tissue damage (33, 34). The irreversible destruction of alveolar walls induces a progressive decline in lung function and increases inflammation (2, 35). Thus, various therapies for S-COPD, including inhaled corticosteroids, bronchodilators (including β2-agonists and anticholinergics), theophyllines, and phosphodiesterase-4 inhibitors, are targeted to reduce airway inflammation (36, 37). Although recent studies have focused on anti-inflammatory treatment for S-COPD (38), the importance of managing the inflammatory features of T-COPD is usually underestimated. Considering that patients with T-COPD exhibited increased inflammatory responses that might be linked with fatty acid pathways and tryptophan catabolism, further studies should focus on treatment strategies to control the associated inflammatory consequences of TB-induced COPD. Following the treatment for TB, aggressive treatments for controlling inflammation should be considered along with the existing COPD treatment modalities, for patients with T-COPD.

This study has some limitations. First, the strengths of associations between the metabolic biomarkers and demographic parameters in healthy controls were not performed due to the absence of pulmonary function tests and cytokine measurements. Correlation analysis of metabolic biomarkers and clinical parameters was conducted only among COPD patients (Supplementary Figure 5). Consequently, we found a positive correlation between oxylipins and IL-6 and CRP levels, while diacylglyceride and colnelenic acid levels were negatively correlated with these parameters. Additionally, positive correlations were identified between lung function scores, such as the rate of FEV_1_ decline and acute exacerbation, and the levels of docosapentanoic acid, 15-HEPE, and long-chain fatty acids were identified. However, the absence of healthy control information resulted in a relatively small slope of the correlations. Second, the metabolomics data included only male patients, since the prevalence of S-COPD is remarkably low in Korean women (39). A validation study would require examinations of females to improve the generalizability of our findings. Moreover, we performed metabolic profiling within one batch and selected metabolites based on strict cut-off values, the FDR-adjusted *P*-value, and fold change, to minimize the output size of raw data and to avoid the batch effect. These processes might limit our ability to detect large changes in metabolites in the direct comparison of patients with S-COPD and those with T-COPD. Further examinations using a validation cohort are required to determine the reproducibility of our findings.

In conclusion, this is the first study to demonstrate increased levels of several inflammatory metabolites in patients with T-COPD compared to those with S-COPD. Lipid and tryptophan metabolism could be potential therapeutic targets for COPD, particularly in T-COPD.

## Data Availability Statement

The metabolomics data are available in the electronic Supplementary Material and at the NIH Common Fund's National Metabolomics Data Repository (NMDR) website (Project ID: PR000943).

## Ethics Statement

The studies involving human participants were reviewed and approved by the institutional review boards (Korea University Guro Hospital: KUGH 13246; Seoul St. Mary's Hospital: KC15OIMI0553; and Chonbuk National University Hospital: 2015-01-018-005), and it fulfilled the tenets of the Declaration of Helsinki. The patients provided written informed consent. The patients/participants provided their written informed consent to participate in this study.

## Author Contributions

DK, JO, and J-YC conceptualized the study. JO, CR, SP, and JS collected samples. DK performed metabolic profiling. The results were interpreted by DK, JO, and J-YC. The original draft preparation was done by DK and JO. J-YC reviewed the manuscript. Funding acquisition was prepared by JO and YC. All authors contributed to the article and approved the submitted version.

## Conflict of Interest

The authors declare that the research was conducted in the absence of any commercial or financial relationships that could be construed as a potential conflict of interest.
