# Supplementary material for: Metabolic Fingerprinting Uncovers the Distinction Between the Phenotypes of Tuberculosis Associated COPD and Smoking-Induced COPD
Source: Front Med (Lausanne). 2021 May 14;8:619077. doi: 10.3389/fmed.2021.619077 (PMC8160120; doi:10.3389/fmed.2021.619077)
Supplement: Supplementary file 1 [file Data_Sheet_1.docx]

Supplementary Material

# Supplementary Data

**Measurements of clinical parameters**

We measured symptoms scores using modified Medical Research Council dyspnoea score, St. George Respiratory Questionnaire (SGRQ) score, and COPD assessment test^10^ score. For pulmonary function test, we performed spirometry, measured the lung volume, and diffusing capacity of the lung for carbon monoxide [DL_CO_].

Blood tests, including tests measuring C-reactive protein (CRP) levels and erythrocyte sedimentation rate (ESR), were performed routinely on the day of enrollment after an overnight fast in the medication-free state. Peripheral whole venous blood samples were collected in tubes containing ethylenediaminetetraacetic acid, and serum was prepared by centrifugation for 10–15 min at 4,500 rpm and stored at -80 °C until analysis. IL-6 levels were measured using enzyme-linked immunosorbent assay kits (IL-6: R&D Systems, Oxford, UK).

The rate of decline in FEV_1_ and acute exacerbation history at one year after enrollment was determined. The rate of decline in FEV_1_ was calculated in the stable state as the change in FEV_1_ from baseline to one year of enrollment. Exacerbation was defined as a sustained deterioration of acute respiratory symptoms beyond the stable state and day-to-day variations that necessitated a change in the regular medication and an unscheduled visit to the hospital. Moderate exacerbation was defined as an increase in symptoms requiring treatment with antibiotics and/or corticosteroids, and severe exacerbation was defined as an increase in symptoms that require hospitalization. Patients who experienced two or more moderate-to-severe exacerbations within one year and with one or more hospitalizations for severe exacerbation were defined as frequent exacerbators.

**Statistical analysis**

The intensity of each ion was normalized, scaled, z-transformed, and aligned according to the retention time using MassHunter Profinder B.08.00 and the Mass Profiler Professional (MPP) software package B.14.9 (Agilent Technologies), to generate a normalized data matrix consisting of the retention time, m/z value, and peak area. Subsequently, a principal component analysis (PCA) was performed using the MPP software for both positive and negative Electrospray ionization (ESI) datasets to determine sample clustering and distinguish ions (filtered by P < 0.05, adjusted for the false discovery rate (FDR) between the healthy controls and patients with COPD). According to the results of the univariate and multivariate analyses, only metabolites obtained in the ESI-negative mode were subjected to further statistical analyses. To reduce the dimensionality from a large number of metabolic features, multivariate analysis (Principal component analysis) were used. Then, univariate analysis to compare metabolites between two different groups (NC vs S-COPD) and (NC vs T-COPD) were performed. The multiple test problem for selecting ions from global metabolomics was corrected by adjusting for the FDR using the Benjamini and Hochberg method, and metabolites with a fold change greater than 2.0 were filtered for identification. The Bonferroni adjustment was performed using the Kruskal-Wallis test to compare the concentrations of metabolites identified in the targeted analyses, and metabolites with fold changes greater than 1.0 were selected. Statistical analyses were performed using GraphPad Prism version 7 (GraphPad Software, Inc., La Jolla, CA, USA) and SPSS 25.0 software (SPSS, Inc.). P-values < 0.05 were considered significant. Network analysis was performed using Cytoscape v. 3.8.0. Selected metabolites with their metabolic information, including the identification number in PubChem, Kyoto Encyclopedia of Genes and Genomes (KEGG) value, and regulation in patients with COPD, were loaded into Cytoscape. Visualization was performed using the yFiles Organic Layout method which is a muli-purpose layout style based on the force-directed layout paradigm.

# Supplementary Figures and Tables

**Table S1.** Limit of detection level of Targeted metabolomics

| Metabolite | Class | LOD (calc.) [µM] | Metabolite | Class | LOD (calc.) [µM] |
| --- | --- | --- | --- | --- | --- |
| C0 | Acylcarnitines | 2.72 | lysoPC a C14:0 | Glycerophospholipids | 0.973 |
| C2 | Acylcarnitines | 0.073 | lysoPC a C16:0 | Glycerophospholipids | 0.12 |
| C3 | Acylcarnitines | 0.066 | lysoPC a C16:1 | Glycerophospholipids | 0.07 |
| C3-DC (C4-OH) | Acylcarnitines | 0.053 | lysoPC a C17:0 | Glycerophospholipids | 0.05 |
| C3-OH | Acylcarnitines | 0.097 | lysoPC a C18:0 | Glycerophospholipids | 0.323 |
| C3:1 | Acylcarnitines | 0.034 | lysoPC a C18:1 | Glycerophospholipids | 0.254 |
| C4 | Acylcarnitines | 0.046 | lysoPC a C18:2 | Glycerophospholipids | 0.1 |
| C4:1 | Acylcarnitines | 0.053 | lysoPC a C20:3 | Glycerophospholipids | 0.2 |
| C5 | Acylcarnitines | 0.057 | lysoPC a C20:4 | Glycerophospholipids | 0.02 |
| C5-DC (C6-OH) | Acylcarnitines | 0.065 | lysoPC a C24:0 | Glycerophospholipids | 1.3 |
| C5-M-DC | Acylcarnitines | 0.092 | lysoPC a C26:0 | Glycerophospholipids | 0.5 |
| C5-OH (C3-DC-M) | Acylcarnitines | 0.08 | lysoPC a C26:1 | Glycerophospholipids | 4 |
| C5:1 | Acylcarnitines | 0.077 | lysoPC a C28:0 | Glycerophospholipids | 0.3 |
| C5:1-DC | Acylcarnitines | 0.076 | lysoPC a C28:1 | Glycerophospholipids | 0.15 |
| C6 (C4:1-DC) | Acylcarnitines | 0.053 | PC aa C24:0 | Glycerophospholipids | 0.1 |
| C6:1 | Acylcarnitines | 0.05 | PC aa C26:0 | Glycerophospholipids | 0.822 |
| C7-DC | Acylcarnitines | 0.069 | PC aa C28:1 | Glycerophospholipids | 0.04 |
| C8 | Acylcarnitines | 0.122 | PC aa C30:0 | Glycerophospholipids | 0.137 |
| C9 | Acylcarnitines | 0.064 | PC aa C30:2 | Glycerophospholipids | 0.006 |
| C10 | Acylcarnitines | 0.097 | PC aa C32:0 | Glycerophospholipids | 0.04 |
| C10:1 | Acylcarnitines | 0.16 | PC aa C32:1 | Glycerophospholipids | 0.06 |
| C10:2 | Acylcarnitines | 0.087 | PC aa C32:2 | Glycerophospholipids | 0.03 |
| C12 | Acylcarnitines | 0.045 | PC aa C32:3 | Glycerophospholipids | 0.008 |
| C12-DC | Acylcarnitines | 0.369 | PC aa C34:1 | Glycerophospholipids | 0.06 |
| C12:1 | Acylcarnitines | 0.121 | PC aa C34:2 | Glycerophospholipids | 0.1 |
| C14 | Acylcarnitines | 0.053 | PC aa C34:3 | Glycerophospholipids | 0.01 |
| C14:1 | Acylcarnitines | 0.082 | PC aa C34:4 | Glycerophospholipids | 0.006 |
| C14:1-OH | Acylcarnitines | 0.015 | PC aa C36:0 | Glycerophospholipids | 0.276 |
| C14:2 | Acylcarnitines | 0.03 | PC aa C36:1 | Glycerophospholipids | 0.03 |
| C14:2-OH | Acylcarnitines | 0.034 | PC aa C36:2 | Glycerophospholipids | 0.15 |
| C16 | Acylcarnitines | 0.018 | PC aa C36:3 | Glycerophospholipids | 0.04 |
| C16-OH | Acylcarnitines | 0.049 | PC aa C36:4 | Glycerophospholipids | 0.04 |
| C16:1 | Acylcarnitines | 0.077 | PC aa C36:5 | Glycerophospholipids | 0.01 |
| C16:1-OH | Acylcarnitines | 0.059 | PC aa C36:6 | Glycerophospholipids | 0.015 |
| C16:2 | Acylcarnitines | 0.023 | PC aa C38:0 | Glycerophospholipids | 0.2 |
| C16:2-OH | Acylcarnitines | 0.032 | PC aa C38:1 | Glycerophospholipids | 0.08 |
| C18 | Acylcarnitines | 0.023 | PC aa C38:3 | Glycerophospholipids | 0.04 |
| C18:1 | Acylcarnitines | 0.205 | PC aa C38:4 | Glycerophospholipids | 0.03 |
| C18:1-OH | Acylcarnitines | 0.102 | PC aa C38:5 | Glycerophospholipids | 0.015 |
| C18:2 | Acylcarnitines | 0.037 | PC aa C38:6 | Glycerophospholipids | 0.02 |
| Ala | Aminoacids | 1 | PC aa C40:1 | Glycerophospholipids | 0.513 |
| Arg | Aminoacids | 0.5 | PC aa C40:2 | Glycerophospholipids | 0.02 |
| Asn | Aminoacids | 1.5 | PC aa C40:3 | Glycerophospholipids | 0.006 |
| Asp | Aminoacids | 1.5 | PC aa C40:4 | Glycerophospholipids | 0.01 |
| Cit | Aminoacids | 1 | PC aa C40:5 | Glycerophospholipids | 0.04 |
| Gln | Aminoacids | 1.5 | PC aa C40:6 | Glycerophospholipids | 0.164 |
| Glu | Aminoacids | 2 | PC aa C42:0 | Glycerophospholipids | 0.05 |
| Gly | Aminoacids | 0.5 | PC aa C42:1 | Glycerophospholipids | 0.008 |
| His | Aminoacids | 0.5 | PC aa C42:2 | Glycerophospholipids | 0.18 |
| Ile | Aminoacids | 0.5 | PC aa C42:4 | Glycerophospholipids | 0.006 |
| Leu | Aminoacids | 1.5 | PC aa C42:5 | Glycerophospholipids | 0.05 |
| Lys | Aminoacids | 0.5 | PC aa C42:6 | Glycerophospholipids | 0.168 |
| Met | Aminoacids | 0.1 | PC ae C30:0 | Glycerophospholipids | 0.129 |
| Orn | Aminoacids | 0.5 | PC ae C30:1 | Glycerophospholipids | 0.02 |
| Phe | Aminoacids | 0.1 | PC ae C30:2 | Glycerophospholipids | 0.57 |
| Pro | Aminoacids | 1 | PC ae C32:1 | Glycerophospholipids | 0.009 |
| Ser | Aminoacids | 1 | PC ae C32:2 | Glycerophospholipids | 0.02 |
| Thr | Aminoacids | 0.5 | PC ae C34:0 | Glycerophospholipids | 0.017 |
| Trp | Aminoacids | 0.5 | PC ae C34:1 | Glycerophospholipids | 0.012 |
| Tyr | Aminoacids | 0.5 | PC ae C34:2 | Glycerophospholipids | 0.01 |
| Val | Aminoacids | 0.5 | PC ae C34:3 | Glycerophospholipids | 0.015 |
| Ac-Orn | Biogenic Amines | 0.2 | PC ae C36:0 | Glycerophospholipids | 0.108 |
| ADMA | Biogenic Amines | 0.08 | PC ae C36:1 | Glycerophospholipids | 0.03 |
| alpha-AAA | Biogenic Amines | 0.4 | PC ae C36:2 | Glycerophospholipids | 0.01 |
| c4-OH-Pro | Biogenic Amines | 0.1 | PC ae C36:3 | Glycerophospholipids | 0.007 |
| Carnosine | Biogenic Amines | 0.1 | PC ae C36:4 | Glycerophospholipids | 0.013 |
| Creatinine | Biogenic Amines | 1 | PC ae C36:5 | Glycerophospholipids | 0.012 |
| DOPA | Biogenic Amines | 0.2 | PC ae C38:0 | Glycerophospholipids | 0.329 |
| Dopamine | Biogenic Amines | 0.1 | PC ae C38:1 | Glycerophospholipids | 0.015 |
| Histamine | Biogenic Amines | 0.01 | PC ae C38:2 | Glycerophospholipids | 0.018 |
| Kynurenine | Biogenic Amines | 0.3 | PC ae C38:3 | Glycerophospholipids | 0.01 |
| Met-SO | Biogenic Amines | 0.3 | PC ae C38:4 | Glycerophospholipids | 0.015 |
| Nitro-Tyr | Biogenic Amines | 0.3 | PC ae C38:5 | Glycerophospholipids | 0.01 |
| PEA | Biogenic Amines | 0.02 | PC ae C38:6 | Glycerophospholipids | 0.03 |
| Putrescine | Biogenic Amines | 0.02 | PC ae C40:1 | Glycerophospholipids | 0.06 |
| SDMA | Biogenic Amines | 0.3 | PC ae C40:2 | Glycerophospholipids | 0.01 |
| Serotonin | Biogenic Amines | 0.03 | PC ae C40:3 | Glycerophospholipids | 0.015 |
| Spermidine | Biogenic Amines | 0.08 | PC ae C40:4 | Glycerophospholipids | 0.117 |
| Spermine | Biogenic Amines | 0.08 | PC ae C40:5 | Glycerophospholipids | 0.006 |
| t4-OH-Pro | Biogenic Amines | 0.1 | PC ae C40:6 | Glycerophospholipids | 0.025 |
| Taurine | Biogenic Amines | 0.8 | PC ae C42:0 | Glycerophospholipids | 0.431 |
| total DMA | Biogenic Amines | 0.1 | PC ae C42:1 | Glycerophospholipids | 0.03 |
| SM (OH) C14:1 | Sphingolipids | 0.025 | PC ae C42:2 | Glycerophospholipids | 0.006 |
| SM (OH) C16:1 | Sphingolipids | 0.012 | PC ae C42:3 | Glycerophospholipids | 0.006 |
| SM (OH) C22:1 | Sphingolipids | 0.015 | PC ae C42:4 | Glycerophospholipids | 0.3 |
| SM (OH) C22:2 | Sphingolipids | 0.01 | PC ae C42:5 | Glycerophospholipids | 0.708 |
| SM (OH) C24:1 | Sphingolipids | 0.01 | PC ae C44:3 | Glycerophospholipids | 0.006 |
| SM C16:0 | Sphingolipids | 0.03 | PC ae C44:4 | Glycerophospholipids | 0.01 |
| SM C16:1 | Sphingolipids | 0.01 | PC ae C44:5 | Glycerophospholipids | 0.186 |
| SM C18:0 | Sphingolipids | 0.07 | PC ae C44:6 | Glycerophospholipids | 0.192 |
| SM C18:1 | Sphingolipids | 0.01 |  |  |  |
| SM C20:2 | Sphingolipids | 0.005 |  |  |  |
| SM C22:3 | Sphingolipids | 0.01 |  |  |  |
| SM C24:0 | Sphingolipids | 0.13 |  |  |  |
| SM C24:1 | Sphingolipids | 0.035 |  |  |  |
| SM C26:0 | Sphingolipids | 0.015 |  |  |  |
| SM C26:1 | Sphingolipids | 0.006 |  |  |  |
| H1 | Sugars | 77.4 |  |  |  |

**Table S2.** Baseline characteristics of healthy subjects

|  | **NC (N=39)** |
| --- | --- |
| **Age, years** | 55.0 (50.0-65.0) |
| **Sex, male** | 39 (100) |
| **BMI, kg/m^2^** | 23.7 (22.3-26.1) |
| **Smoking amount (pack-year)** | 0.0 (0.0-17.0) |

**Notes:** Data are presented as median (interquartile range) for continuous variables and number (percentage) for categorical variables.

**Abbreviations:** BMI, body mass index; NC, normal control

**Table S3.** Metabolites observed using targeted and global metabolomics profiling

| **Metabolite** | **Class** | **T-COPD vs NC** | | **S-COPD vs NC** | |
| --- | --- | --- | --- | --- | --- |
|  |  | **Fold change** | **p-value** | **Fold change** | **p-value** |
| C12 | Acylcarnitines | 1.889 | 2.5755E-10 | 1.7815 | 2.5554E-11 |
| C141 | Acylcarnitines | 2.1289 | 8.0279E-10 | 2.0571 | 2.0208E-10 |
| C161 | Acylcarnitines | 1.8337 | 1.9668E-09 | 1.9556 | 1.6633E-10 |
| C10 | Acylcarnitines | 1.9341 | 3.8618E-09 | 1.8095 | 1.289E-09 |
| C121 | Acylcarnitines | 1.8973 | 7.2488E-09 | 1.8969 | 1.5373E-09 |
| C8 | Acylcarnitines | 1.7185 | 2.1473E-08 | 1.6602 | 2.225E-09 |
| C14 | Acylcarnitines | 1.4571 | 1.0948E-07 | 1.3826 | 4.7718E-06 |
| C16 | Acylcarnitines | 1.3575 | 1.3388E-07 | 1.4352 | 2.5978E-09 |
| C181 | Acylcarnitines | 1.4387 | 2.1473E-07 | 1.5144 | 7.1394E-09 |
| C142 | Acylcarnitines | 1.699 | 1.3131E-06 | 1.6386 | 8.8834E-08 |
| C7-DC | Acylcarnitines | 1.8958 | 4.0679E-06 | 1.9596 | 9.922E-08 |
| C101 | Acylcarnitines | 1.4649 | 0.000014934 | 1.4578 | 2.2256E-06 |
| C182 | Acylcarnitines | 1.3537 | 0.000042431 | 1.4427 | 6.3679E-06 |
| C2 | Acylcarnitines | 1.3436 | 0.000061632 | 1.3934 | 5.4068E-06 |
| PC ae C425 | Glycerophospholipids | 1.2609 | 0.000081067 | 1.3652 | 1.4566E-06 |
| C6 C41-DC | Acylcarnitines | 1.3392 | 0.00014256 | 1.3484 | 0.00010104 |
| Glu | Amino acids | 1.3562 | 0.00049406 | 1.3637 | 0.000093536 |
| PC ae C405 | Glycerophospholipids | 1.266 | 0.0014411 | 1.527 | 6.8658E-06 |
| PC aa C321 | Glycerophospholipids | 1.4079 | 0.0016524 | 1.5545 | 0.00031406 |
| PC ae C424 | Glycerophospholipids | 1.3005 | 0.0018242 | 1.5112 | 5.3222E-06 |
| C9 | Acylcarnitines | 1.3351 | 0.0019484 | 1.2528 | 0.0098145 |
| PC ae C420 | Glycerophospholipids | 1.15 | 0.0020108 | 1.2493 | 8.5572E-06 |
| PC ae C403 | Glycerophospholipids | 1.3346 | 0.0023567 | 1.663 | 0.000012528 |
| C162 | Acylcarnitines | 1.6451 | 0.0024062 | 1.5903 | 0.0014071 |
| Spermidine | Biogenic amines | 1.322 | 0.0026633 | 1.3214 | 0.016836 |
| lysoPC a C161 | Glycerophospholipids | 1.2851 | 0.002721 | 1.4691 | 5.184E-07 |
| C142-OH | Acylcarnitines | 1.3492 | 0.0032184 | 1.2644 | 0.029303 |
| PC aa C404 | Glycerophospholipids | 1.188 | 0.0035239 | 1.3476 | 0.00015661 |
| PC aa C425 | Glycerophospholipids | 1.2787 | 0.0041444 | 1.4804 | 0.00007 |
| PC aa C402 | Glycerophospholipids | 1.3153 | 0.0042888 | 1.7757 | 0.000021256 |
| PC aa C424 | Glycerophospholipids | 1.5822 | 0.0043815 | 2.0511 | 0.000089924 |
| PC aa C403 | Glycerophospholipids | 1.2569 | 0.0050839 | 1.5704 | 0.000021548 |
| PC ae C422 | Glycerophospholipids | 1.2427 | 0.0050846 | 1.5659 | 0.000025888 |
| PC ae C402 | Glycerophospholipids | 1.1906 | 0.0051982 | 1.4116 | 0.000063736 |
| PC ae C404 | Glycerophospholipids | 1.2266 | 0.0054366 | 1.4656 | 8.5716E-06 |
| C18 | Acylcarnitines | 1.2154 | 0.0091465 | 1.3164 | 0.0011929 |
| PC aa C383 | Glycerophospholipids | 1.1556 | 0.010186 | 1.1478 | 0.031356 |
| C102 | Acylcarnitines | 1.1597 | 0.01027 | 1.1644 | 0.0021388 |
| PC ae C443 | Glycerophospholipids | 1.5847 | 0.010404 | 2.2065 | 0.000084434 |
| C141-OH | Acylcarnitines | 1.6604 | 0.018172 | 1.606 | 0.022566 |
| PC ae C383 | Glycerophospholipids | 1.1962 | 0.022665 | 1.474 | 0.00016271 |
| PC aa C426 | Glycerophospholipids | 1.2021 | 0.025862 | 1.333 | 0.00057059 |
| PC ae C380 | Glycerophospholipids | 1.1527 | 0.027348 | 1.3259 | 0.00041959 |
| PC aa C422 | Glycerophospholipids | 1.4057 | 0.030501 | 1.7888 | 0.0016146 |
| PC aa C300 | Glycerophospholipids | 1.1589 | 0.032255 | 1.2472 | 0.0057356 |
| PC ae C381 | Glycerophospholipids | 1.3326 | 0.032544 | 1.7354 | 0.00090278 |
| PC ae C421 | Glycerophospholipids | 1.1853 | 0.033453 | 1.4081 | 0.00077086 |
| PC aa C381 | Glycerophospholipids | 1.1923 | 0.033751 | 1.3044 | 0.0011312 |
| Tyr | Amino Acids | 1.1078 | 0.034363 | 1.1704 | 0.0020577 |
| PC ae C423 | Glycerophospholipids | 1.1661 | 0.039261 | 1.3737 | 0.00093485 |
| PC aa C401 | Glycerophospholipids | 1.1655 | 0.040322 | 1.329 | 0.00014328 |
| C41 | Acylcarnitines | 1.2385 | 0.049281 | 1.2135 | 0.032826 |
| lysoPC a C182 | Glycerophospholipids | 0.70291 | 7.9159E-06 | 0.77765 | 0.00018226 |
| H1 | Monosaccharides | 0.7411 | 0.00011114 | 0.70993 | 0.000023466 |
| PC aa C342 | Glycerophospholipids | 0.8673 | 0.00095784 | 0.89256 | 0.0035273 |
| Pro | Amino acids | 0.87776 | 0.0017567 | 0.86275 | 0.0030578 |
| alpha-AAA | Biogenic amines | 0.42142 | 0.0030451 | 0.46063 | 0.0013084 |
| PC aa C362 | Glycerophospholipids | 0.89955 | 0.019999 | 0.896 | 0.017452 |
| Ala | Amino acids | 0.85022 | 0.0036924 | 0.8649 | 0.010553 |
| C31 | Acylcarnitines |  |  | 0.60372 | 0.0024509 |
| C3-DC C4-OH | Acylcarnitines |  |  | 1.3248 | 0.011787 |
| lysoPC a C140 | Glycerophospholipids |  |  | 1.1142 | 0.000077795 |
| lysoPC a C160 | Glycerophospholipids |  |  | 1.2021 | 0.00034582 |
| lysoPC a C170 | Glycerophospholipids |  |  | 1.1763 | 0.0087027 |
| lysoPC a C180 | Glycerophospholipids |  |  | 1.2022 | 0.0058463 |
| lysoPC a C240 | Glycerophospholipids |  |  | 1.1685 | 0.02382 |
| lysoPC a C280 | Glycerophospholipids |  |  | 1.8943 | 0.033613 |
| lysoPC a C281 | Glycerophospholipids |  |  | 1.8241 | 0.01241 |
| Met-SO | Biogenic amines |  |  | 1.5794 | 0.00029857 |
| PC aa C260 | Glycerophospholipids |  |  | 1.1209 | 0.024016 |
| PC aa C320 | Glycerophospholipids |  |  | 1.1383 | 0.023815 |
| PC aa C323 | Glycerophospholipids |  |  | 1.3087 | 0.016298 |
| PC aa C360 | Glycerophospholipids |  |  | 1.1536 | 0.035839 |
| PC aa C366 | Glycerophospholipids |  |  | 1.4019 | 0.0062555 |
| PC aa C420 | Glycerophospholipids |  |  | 1.2033 | 0.039279 |
| PC aa C421 | Glycerophospholipids |  |  | 1.3246 | 0.040438 |
| PC ae C301 | Glycerophospholipids |  |  | 1.4406 | 0.0057908 |
| PC ae C302 | Glycerophospholipids |  |  | 1.1351 | 0.0037123 |
| PC ae C321 | Glycerophospholipids |  |  | 1.1496 | 0.021928 |
| PC ae C322 | Glycerophospholipids |  |  | 1.1945 | 0.0094533 |
| PC ae C360 | Glycerophospholipids |  |  | 1.1302 | 0.033136 |
| PC ae C361 | Glycerophospholipids |  |  | 1.1865 | 0.0077083 |
| PC ae C382 | Glycerophospholipids |  |  | 1.2879 | 0.0027314 |
| PC ae C401 | Glycerophospholipids |  |  | 1.2323 | 0.0078519 |
| PC ae C406 | Glycerophospholipids |  |  | 1.1446 | 0.02286 |
| PC ae C421 | Glycerophospholipids |  |  | 1.4081 | 0.00077086 |
| PC ae C444 | Glycerophospholipids |  |  | 1.4518 | 0.0006401 |
| SM C261 | Glycerophospholipids |  |  | 1.3596 | 0.014682 |
| Asp | Amino acids | 1.2196 | 0.016775 |  |  |
| C12-DC | Acylcarnitines | 1.1003 | 0.024043 |  |  |
| Kynurenine | Biogenic amines | 1.1555 | 0.00039572 |  |  |
| lysoPC a C204 | Glycerophospholipids | 0.85619 | 0.049607 |  |  |
| PC aa C364 | Glycerophospholipids | 0.8998 | 0.031666 |  |  |
| PC aa C406 | Glycerophospholipids | 1.1902 | 0.016125 |  |  |
| PC ae C445 | Glycerophospholipids | 1.1096 | 0.027861 |  |  |
| Serotonin | Biogenic amines | 0.75599 | 0.002111 |  |  |
| Thr | Amino acids | 0.89534 | 0.034943 |  |  |
| Myristoleic acid | Fatty acids | 2.5872505 | 4.02E-11 | 2.5643601 | 1.12E-10 |
| Palmitic acid | Fatty acids | 2.1976414 | 3.74E-07 | 2.1628566 | 5.85E-07 |
| Oleic acid | Fatty acids | 2.3216546 | 2.78E-08 | 2.2500045 | 9.53E-08 |
| Ethyl Arachidonate | Fatty acid esters | 2.064736 | 6.03E-06 | 2.0095186 | 1.20E-05 |
| PGE | Eicosanoids | 2.2050905 | 3.29E-07 | 2.2478914 | 9.53E-08 |
| Arachidonic acid | Fatty acids | 2.002016 | 2.13E-05 |  |  |
| 9Z-Eicosenoic acid | Fatty acids | 2.0763113 | 5.01E-06 |  |  |
| Docosapentaenoic acid | Fatty acids | 2.1420026 | 1.25E-06 |  |  |
| Dihomo-alpha-linolenic acid | Fatty acids | 2.1537056 | 9.90E-07 |  |  |
| DiHomo Linoleic acid | Fatty acids | 2.0840206 | 4.27E-06 |  |  |
| 5-Oxooctadecanoic acid | Fatty acids | 2.2185419 | 2.62E-07 |  |  |
| 2-Hydroxymyristic acid | Fatty acids | 2.3615763 | 1.53E-08 |  |  |
| 15(S)-HETrE | Medium-chain hydroxy acids | 2.1579776 | 9.18E-07 |  |  |
| 3-Oxohexadecanoic acid | Fatty acids | 2.3318772 | 2.36E-08 |  |  |
| DG(33:3) | Lineolic acids | -2.0186517 | 1.54E-05 |  |  |
| 3-Sulfodeoxycholic acid | Bile acids | 2.0665345 | 5.90E-06 |  |  |
| 15-HEPE | Eicosanoids | 2.1201723 | 1.97E-06 |  |  |
| Inosine | Purine nucleosides |  |  | -2.0192366 | 9.63E-06 |
| (2'E,4'Z,7'Z,8E)-Colnelenic acid | Fatty acids |  |  | 2.088976 | 2.65E-06 |
| Pregnenolone | Pregnane steroids |  |  | 2.2173703 | 1.72E-07 |
| 11b,21-Dihydroxy-5b-pregnane-3,20-dione | Pregnenolone |  |  | 2.1234384 | 1.31E-06 |
| 4-cholesten-7α,12α,24-triol-3-one | Bile acids |  |  | 2.0558696 | 4.90E-06 |
| DG(38:8) | Lineolic acids |  |  | 2.117313 | 1.46E-06 |
| PE(29:1) | Glycerophospholipids |  |  | 2.0679817 | 4.01E-06 |
| PC(28:2) | Glycerophospholipids |  |  | 2.435124 | 1.43E-09 |
| PA(36:8) | Glycerophospholipids |  |  | 2.1300838 | 1.14E-06 |

**Table S4.** List of metabolites observed using global metabolomics profiling

| **m/z** | **Retention Time** | **HMDB ID** | **Chemical formulae** | **Detected ion** | **MSI level** | **Regulation in COPD** | **Metabolite** | **FDR-adjusted p-value** |
| --- | --- | --- | --- | --- | --- | --- | --- | --- |
| 304.2341 | 13.432 | 0001043 | C20H32O2 | [M+H]- | 1 | up | Arachidonic acid | 2.13E-05 |
| 310.2802 | 14.058 | 0002231 | C20H38O2 | [M+H]- | 1 | up | 11Z-Eicosenoic acid | 5.01E-06 |
| 330.2544 | 13.617 | 0001976 | C22H34O2 | [M+H]- | 2 | up | Docosapentaenoic acid (22n-6) | 1.25E-06 |
| 306.249 | 13.617 | 0010378 | C20H34O2 | [M+H]- | 1 | up | 5,8,11-Eicosatrienoic acid | 9.90E-07 |
| 308.2653 | 13.828 | 0005060 | C20H36O2 | [M+H]- | 1 | up | 11Z,14Z-Eicosa-11,14-dienoic acid | 4.27E-06 |
| 298.2467 | 13.167 | 0034074 | C18H34O3 | [M+H]- | 2 | up | 5-Oxooctadecanoic acid | 2.62E-07 |
| 244.2019 | 12.415 | 0002261 | C14H28O3 | [M+H]- | 1 | up | 2-Hydroxymyristic acid | 1.53E-08 |
| 322.2121 | 13.322 | 0005050 | C20H32O4 | [M+H]- | 1 | up | 15(S)-HETrE | 9.18E-07 |
| 270.2148 | 12.614 | 0010733 | C16H30O3 | [M+H]- | 2 | up | 3-Oxohexadecanoic acid | 2.36E-08 |
| 576.4658 | 14.116 | 0055996 | C36H64O5 | [M+H]- | 3 | down | DG(33:3) | 1.54E-05 |
| 458.2363 | 13.727 | 0002504 | C23H38O7S | [M+H]- | 1 | up | 3-Sulfodeoxycholic acid | 5.90E-06 |
| 318.2206 | 12.602 | 0010209 | C20H30O3 | [M+H]- | 1 | up | 15-HEPE | 1.97E-06 |
| 268.0774 | 1.789 | 0000195 | C10H12N4O5 | [M+H]- | 1 | down | Inosine | 9.63E-06 |
| 292.2003 | 12.258 | 0030996 | C18H28O3 | [M+H]- | 2 | up | (2'E,4'Z,7'Z,8E)-Colnelenic acid | 2.65E-06 |
| 298.244 | 12.459 | 0000253 | C21H32O2 | [M+H2O+H]- | 1 | up | Pregnenolone | 1.72E-07 |
| 300.2629 | 12.697 | 00062549 | C18H36O3 | [M+H]- | 2 | up | 2-Hydroxystearic acid | 1.59E-07 |
| 324.2209 | 13.656 | 0006757 | C21H32O4 | [M+H]- | 2 | up | 11b,21-Dihydroxy-5b-pregnane-3,20-dione | 5.24E-06 |
| 432.3248 | 13.729 | 0062399 | C27H44O4 | [M+H]- | 2 | up | 4-cholesten-7α,12α,24-triol-3-one | 1.31E-06 |
| 226.1892 | 12.796 | 0002000 | C14H26O2 | [M+H]- | 2 | up | Myristoleic acid | <0.05 |
| 256.2388 | 13.657 | 0000220 | C16H32O2 | [M+H]- | 1 | up | Palmitic acid |  |
| 282.2526 | 13.731 | 0000207 | C18H34O2 | [M+H]- | 1 | up | Oleic acid |  |
| 332.2689 | 13.740 | 0062725 | C22H36O2 | [M+H]- | 2 | up | Ethyl Arachidonate |  |
| 350.2367 | 13.731 | 0002664 | C20H30O5 | [M+H]- | 2 | up | PGE |  |

**Table S5**. Targeted metabolites associated T-COPD compared to S-COPD

| **metabolites** | **Univariate analysis** | | | **Multivariate analysis*** | |
| --- | --- | --- | --- | --- | --- |
|  | **OR (95% CI)** | ***P*-value** | **OR (95% CI)** | | ***P*-value** |
| **Asparagine** | 0.952 (0.726-0.978) | <0.001 | 0.959 (0.923-0.997) | | 0.036 |
| **Aspartate** | 1.029 (1.006-1.053) | 0.015 | 1.038 (1.007-1.070) | | 0.016 |
| **Histidine** | 0.961 (0.937-0.986) | 0.002 | - | | - |
| **Kyneurenine** | 1.699 (1.014-2.848) | 0.044 | - | | - |
| **Serotonin** | 0.085 (0.015-.0.479) | 0.005 | 0.060 (0.005-0.692) | | 0.024 |
| **Glutamine** | 0.995 (0.991-0.999) | 0.026 | - | | - |
| **Glycerine** | 0.992 (0.985-0.999) | 0.032 | - | | - |
| **lysophosphatidylcholine a C14:0** | 0.567 (0.357-0.902) | 0.017 | - | | - |

**Notes:** *Variables with a P-value <0.05 on univariate analysis and demographic factors including age, bmi, underlying diseases and smoking history were analyzed using multivariate logistic regression analysis.


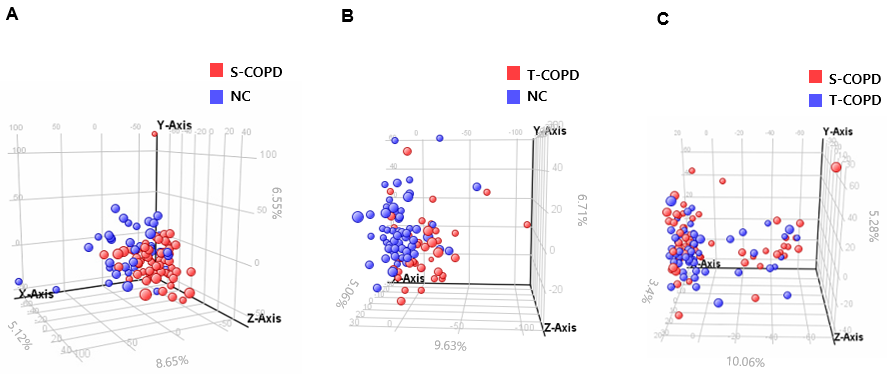


**Figure S1.** Score plots of principle component analysis

Metabolic profiling was performed using LC-QTOF-MS. The three dimensional principal component analysis (PCA) showed metabolic patterns of **A** NC vs. S-COPD, **B** NC vs T-COPD**, and C** S-COPD vs. T-COPD. In **A** and **B**, blue and red spheres indicate normal control and patients with COPD, respectively. In **C**, blue and red spheres indicate S-COPD and T-COPD, respectively.


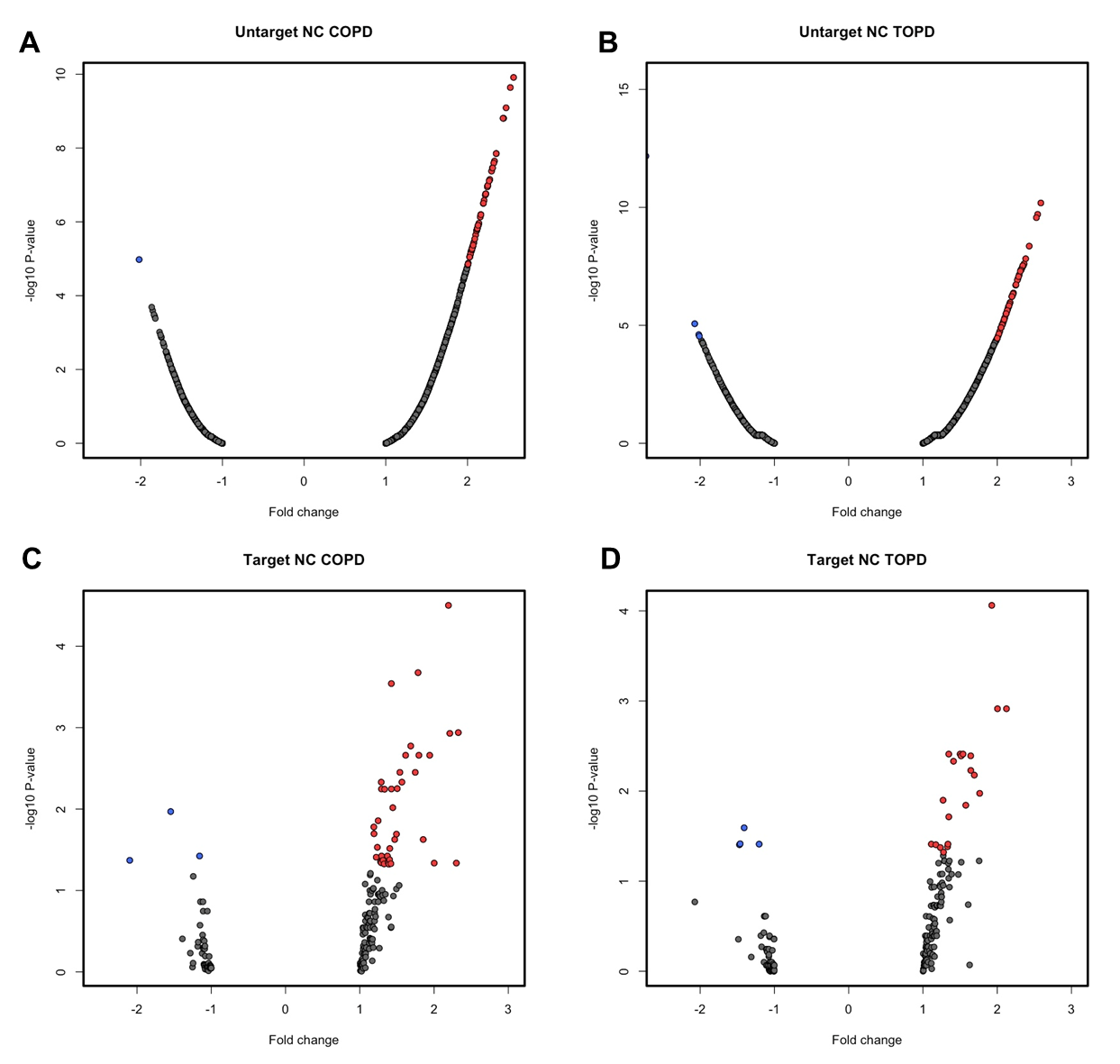


**Figure S2.** Volcano plots of metabolomics profiling

**A** and **B** shows the statistical results of global metabolomics profiling in the comparison of NC vs S-COPD group, and NC vs T-COPD group, respectively. Compounds less than 0.05 of FDR-adjusted p-value and fold change greater than 2.0 were selected. Blue circles indicate down-regulation of metabolites in the patients (S-COPD or T-COPD) compared to NC and red circles indicate up-regulated metabolites in the patients. **C** and **D** are the volcano plots of targeted metabolomics profiling. **C** shows the comparison between NC vs S-COPD and **D** shows the comparison of NC vs T-COPD Compound less than 0.05 of FDR-adjusted p-value with fold change greater than 1.0 were selected. Blue circles indicate down-regulation of metabolites in the patients (S-COPD or T-COPD) compared to NC and red circles indicate up-regulated metabolites in the patients.

**Figure S3.** Changes of metabolites along the disease severity

Whiskers in the boxplots represent the range of the minimum and maximum concentrations of serum metabolites within a population. S-COPD_0 and T-COPD_0 indicate FEV1 < 50% and S-COPD_1 and T-COPD_1 indicate FEV1 > 50%. Asterisk represents p-value less than 0.05 between severity score 0 and 1


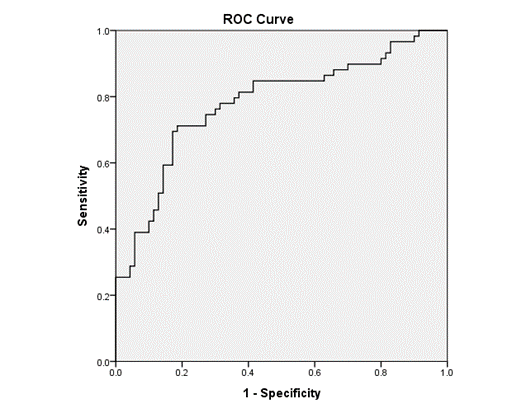


**Figure S4.** ROC curve for diagnosis of T-COPD

The AUC of T-COPD diagnosis with aspartate, asparagine and serotonin was 0.780.


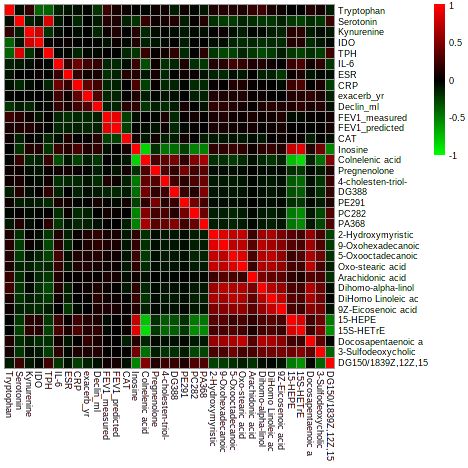


**Figure S5.** Correlation analysis of metabolites with clinical parameters

Pearson’s correlation analysis was performed to investigate the association between the circulating metabolites and clinical parameters of COPD patients. Red and green squares indicate positive and negative correlations, respectively.
